# Real-time forecasting of emergency department arrivals using prehospital data

**DOI:** 10.1186/s12873-019-0256-z

**Published:** 2019-08-05

**Authors:** Andreas Asheim, Lars P. Bache-Wiig Bjørnsen, Lars E. Næss-Pleym, Oddvar Uleberg, Jostein Dale, Sara M. Nilsen

**Affiliations:** 1 Center for Health Care Improvement, St. Olav’s Hospital HF, Trondheim University Hospital, Trondheim, Norway; 2 Department of Mathematical Sciences, Norwegian University of Science and Technology, Trondheim, Norway; 3 Department of Emergency Medicine and Pre-hospital Services, St. Olav’s Hospital HF, Trondheim University Hospital, Trondheim, Norway; 4 Department of Circulation and Medical Imaging, Norwegian University of Science and Technology, Trondheim, Norway; 5 Department of Research and Development, Norwegian Air Ambulance Foundation, Drøbak, Norway

**Keywords:** Emergency department crowding, Prediction, Decision support

## Abstract

**Background:**

Crowding in emergency departments (EDs) is a challenge globally. To counteract crowding in day-to-day operations, better tools to improve monitoring of the patient flow in the ED is needed. The objective of this study was the development of a continuously updated monitoring system to forecast emergency department (ED) arrivals on a short time-horizon incorporating data from prehospital services.

**Methods:**

Time of notification and ED arrival was obtained for all 191,939 arrivals at the ED of a Norwegian university hospital from 2010 to 2018. An arrival notification was an automatically captured time stamp which indicated the first time the ED was notified of an arriving patient, typically by a call from an ambulance to the emergency service communication center. A Poisson time-series regression model for forecasting the number of arrivals on a 1-, 2- and 3-h horizon with continuous weekly and yearly cyclic effects was implemented. We incorporated time of arrival notification by modelling time to arrival as a time varying hazard function. We validated the model on the last full year of data.

**Results:**

In our data, 20% of the arrivals had been notified more than 1 hour prior to arrival. By incorporating time of notification into the forecasting model, we saw a substantial improvement in forecasting accuracy, especially on a one-hour horizon. In terms of mean absolute prediction error, we observed around a six percentage-point decrease compared to a simplified prediction model. The increase in accuracy was particularly large for periods with large inflow.

**Conclusions:**

The proposed model shows increased predictability in ED patient inflow when incorporating data on patient notifications. This approach to forecasting arrivals can be a valuable tool for logistic, decision making and ED resource management.

**Electronic supplementary material:**

The online version of this article (10.1186/s12873-019-0256-z) contains supplementary material, which is available to authorized users.

## Background

Counteracting crowding in emergency departments (EDs) is a global challenge. Inadequate handling of crowding may lead to suboptimal ED functioning [1], which in turn might be linked to negative patient outcomes [2, 3]. Planning an adequate response to potential crowding may require, among other, knowledge about patient input. Arrival rates of ED patients is characterized by large daily, weekly and seasonal variations, along with a degree of inherent unpredictability [4], and large transient volumes of arriving patients (input) has been identified as a cause of crowding, along with delays in throughput and output [5].

There exists a large literature on forecasting ED arrivals [6], but much of the variation in ED arrivals remain unaccounted for. This is more pronounced on short time spans: The forecasted daily number of arrivals at a typical ED show a mean absolute percentage error (MAPE) of 10% [7, 8], while for hourly admissions this typically lies around 50% [7].

In terms of planning levels, forecasts of daily arrivals are excellent for aiding medium-term planning, e.g. assigning rotas. Tactical planning, e.g., deciding when to contact staff on call, requires situational awareness and more fine-grained information than what is provided by daily totals. Prior notice of when the bulk of the patients arrive is of importance. Implementing such a forecast in a real-time monitoring system is a way of providing support for decisions and give the medical and administrative staff a common situational awareness. However, a forecast need to reflect available information and provide sufficient accuracy. Existing methods do not satisfy these two criteria.

The primary objective of this study was the development of a real-time system for decision support that could forecast ED arrivals on a short time-horizon, incorporating data from prehospital services. A secondary objective was to assess whether such an approach could improve accuracy over existing methods.

## Methods

### Data and setting

St. Olav’s University Hospital is an academic teaching hospital located in Trondheim, Norway, with more than 20,000 ED arrivals per year [9]. Data were obtained from the local ED database (Akuttdatabasen Version 1.6.3.31495, Helse-Vest IKT, Stavanger, Norway). We extracted time of arrival for all 191,939 patients arriving from January 1st 2010 to December 31st 2018. A subset of this data, the 23,757 arrivals between January 1st 2017 and December 31st 2017, was used as validation set and were withheld from the training set, which then includes 168,182 arrivals.

The database did not include patients eligible for bypass protocols, for example, percutaneous coronary intervention and “fast-track” hip fractures. Obstetrics and gynecology, ear, nose, and throat, orthopedic, and pediatric patients are typically seen 24/7 at separate EDs or outpatient clinics [10]. The clinical setting is previously described [9, 10]. One distinct characteristic of the Norwegian health care system is that the general practitioners (GP) are considered the “gate keepers” who refer patients to the EDs.

### Time registrations

For this study we used both time of patient record creation, which we refer to as time of notification, and time of arrival. When a patient arrived at the ED without prior notice, the patient record would be created at the time of arrival. Otherwise, prehospital services would have been in contact with the ED via the emergency service communication center (EMCC) prior to arrival. This would result in a patient record being created in the ED database, and the time of creation would be logged. We refer to these patients as pre-reported. All time registrations were recorded with precision in minutes. In practice, many of the time registrations could be expected to be rounded to the nearest 10 min [11]. Whenever a patient record was created less than 10 min before arrival, we marked the arrival as not pre-reported.

### Software

All analyses were done using RStudio (version 1.1.442, Free Software Inc., Boston MA).

### Forecasting model

We used a Poisson time-series regression model fitted with the *glm* function in R for forecasting arrivals on a short time-horizon. Daily and weekly variation was modelled using a trigonometric polynomial with a period of 7 days, corrected for daylight savings time. This approach captures rapid variations within each day in a continuous way, as well as differences between weekdays. Seasonal variation was modelled using a trigonometric polynomial with a period of 365 days. A linear and quadratic term was introduced to account for a possible long-term temporal trend, capturing a potential increase in arrivals, and indicator variables were introduced to capture the effect of holidays and days after holidays. The number of terms to include in the modelling of seasonal and weekly variation was chosen by stepwise inclusion of terms, stopping when the Akaike information criterion (AIC) for the method was minimized.

A time series was assembled by sampling the data on regular intervals in the study period. For any time-point in the study period, we could compute the state of the ED, in particular the number of arrivals within a forecasting horizon of 1, 2, and 3 h. We sampled the data on five-minute intervals, such that an updated forecast of these numbers could be displayed with such regularity. In addition to the number of arrivals in the following hours, the number of arrivals in the previous 1, 2, and 3 h were used as a correction to capture the effect of periods with persistent, large inflow. Examples of this could be flu season or icy conditions.

We also computed the number of pre-reported patients who had not yet arrived. We let n(t) denote the number of such patients at time t, and t_j_, j = 1, ..., n(t), the time since each patient record was created. If any patient was marked as pre-reported for more than 6 h, this patient was left out of the following computation, by the assumption that these were probably expected to arrive the following day. To incorporate this data into the model, we used an approximate hazard function, i.e., the probability of an arrival, given that the patient had not yet arrived, as a predictor. The resulting expected number of arrivals was then used as a predictor in the model. Restricting the hazard function to a third-degree polynomial meant that n(t), ∑t_j_, ∑t_j_^2^ and ∑t_j_^3^ were used as predictors in the model. Details can be found in Additional file 1.

### Error analysis

For all the error analyses the last full year of the data was used. This data was not used in the training of the model (cf. [6]).

As baseline for comparison we used a Poisson regression model, similar to what has been suggested in literature [4], using arrivals categorized by months, days and hours as predictors. The same corrections as in the proposed method were used in the baseline method (holidays, day after holiday, arrivals in the preceding hour and long-time trend). This method only gave one updated forecast per hour and took no account of time of patient notification. We also tested the proposed model with predictors based on time of notification left out. Comparing this reduced model with the baseline captured the effect of the more detailed description of time variation.

We used the mean absolute percentage errors (MAPE) for the methods as an overall accuracy measure. We also compared the methods in their accuracy when forecasting periods of large influx by plotting the mean signed error of the methods against the actual inflow.

## Results

A large part of the population (36%) had less than 10 min from time of patient notification to arrival and were marked as not pre-reported. The average time from patient notification to arrival was 58 min, while less than 1% had 5 h or more. The distribution of time from notification to arrival is displayed in Additional file 2.

**Fig. 1:**
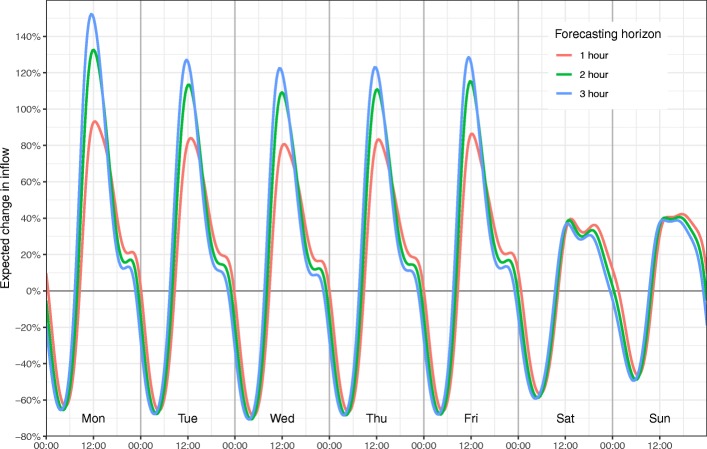
Weekly variation. Displayed in terms of percentage expected change in inflow within 1, 2 and 3 h

Fitting the method on the training data, resulted in 48 Fourier terms to capture the variation through the week, and 16 terms through the year. The resulting cyclic patterns throughout the week can be seen in the Fig. 1, which shows the computed relative change in the expected number of patient arrivals within a 1-,2-, and 3-h forecasting horizon, as a function of time of day and weekday. We observe that the model captures a sharp rise in patient input at the beginning of each day, with a peak around mid-day. Yearly patterns are shown in Additional file 3.

**Fig. 2:**
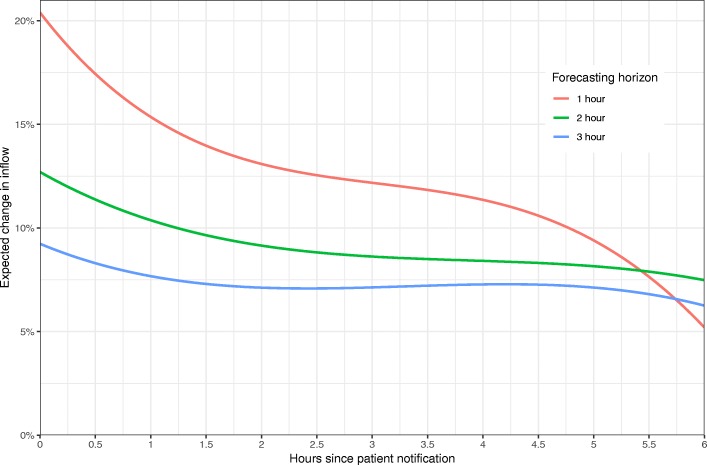
Percentage change in expected number of patients whenever one patient is reported while not arrived. Displayed as a function of time since patient record creation

The impact of patient notification on the forecasted number of patients is shown in Fig. 2, showing the computed relative change in the expected number of patient arrivals within a 1-,2-, and 3-h forecasting horizon as a function of time since a patient notification. Per extra pre-reported patient, we see a 20% increase in the expected number of arrivals in the following hour, which decreases to about 5% when the time since patient notification was 6 h. The impact of a pre-reported patient was markedly lower when forecasting on longer time horizons, showing around 10% higher inflow per pre-reported patient. For the 2- and 3-h forecasts, the time since notification had less impact.

**Fig. 3:**
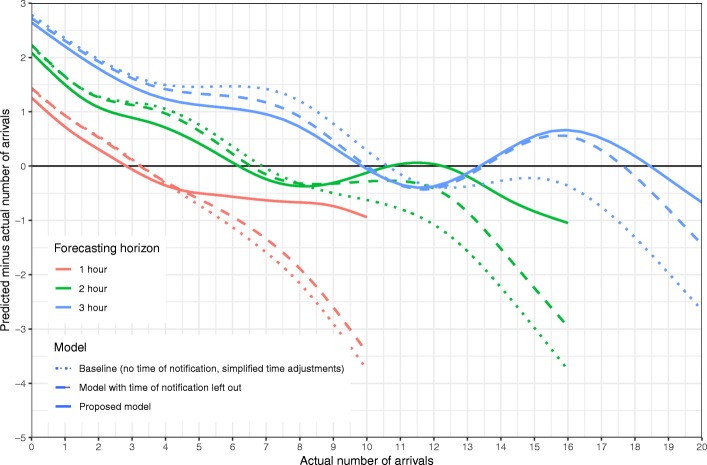
Average forecasting error as a function of number of arrivals. Continuous curves are the errors for the proposed model, forecasting on 1-, 2- and 3-h horizon. Dashed lines are errors of the model when not taking time of patient notification into account, dotted lines are errors of the baseline model, which takes neither patient notification, nor continuous time adjustment into account

Measuring the MAPE of the baseline method, which took no account of patient notification, nor included continuous time adjustments, gave 57, 43 and 36% on 1-, 2- and 3- h forecasting horizons respectively. The full model with time of notification left out, improved each of these measures by three percentage points. Using the full model, which included pre-reported patients and continuous time adjustments, further reduced the MAPE to 52, 38 and 31%. Figure 3 shows the average signed error of the model as well as the two comparisons as a function of inflow, for the forecasts of number of patients within a 1-, 2- and 3-h forecasting horizon. We observe that the models tended to underestimate the inflow as the actual number of arrivals increased. The effect of including pre-reported patients in the model primarily increased the accuracy when the inflow was higher. When 10 patients arrived within 1 h, we see that the proposed model underestimated with less than 2 patients on average, while not taking pre-reported patients into account resulted in an underestimation of 4 patients. For the 2- and 3-h forecasting horizons we see that including pre-reported patients gave a similar effect, although less pronounced. On the longer forecasting horizon, the more detailed description of time-variation also had an impact on accuracy.

## Discussion

Crowding in EDs is a challenge worldwide which may lead to negative patient outcomes [2, 3]. A tool for predicting arrivals has the potential to improve situational awareness and contribute to counteracting crowding problems. Knowledge of when the bulk of the patients arrive, not just daily aggregates, is then of importance. The model proposed can be used as an objective tool for the hospital to allocate and activate resources, e.g., calling in nurses and physicians, discharge patients from the wards, open additional patient beds, communicate with admitting physicians about alternative logistical options for their patients, and admit appropriate patients to units outside the hospital run by the municipalities. In practice, this forecasting model can be implemented in daily clinical use, for example by expanding existing decision support systems which track the patients in the ED. Adding an estimate of the number of patients to arrive within some time span requires little investment if updated information is available.

Existing literature on forecasting ED arrivals recommend calendar variables like time-of the day, day-of-the-week and month as predictors [6]. In addition, some effort has been put into using ambient variables, like weather and amount of daylight [12], internet searches [13], etc. Various forecasting models have been attempted, such as exponential smoothing [14], generalized linear models (GLM), auto-regressive integrated moving average (ARIMA) models, and artificial neural networks [15]. Still, parts of the variation in ED arrivals remain unaccounted for and is likely due to chance. This is more evident on short time spans and is not likely to be solved by adding more ambient variables. By incorporating time of patient notification, we effectively recast the problem of forecasting as a problem of predicting time of arrival for those patients whose arrival is imminent. Thereby the effect of chance is lessened, the forecasts are more accurate and better reflect available information. The large variations that happen within a short time-frame necessitates continuous handling of the data for the model to increase situational awareness. One strength of our proposed model is that it takes precise time of arrival into account and output forecasts that can, in principle, be continuously updated.

Although we observe a clear improvement in the mean absolute percentage error (MAPE), which is one standard error measure for forecasting methods, the MAPE is still over 50%. It should be noted that this error measure is sensitive to periods of small inflow. We suggest, rather than a single number measure, to considering the forecasting error in terms of actual inflow. A period of large inflow is more important to detect in advance. For the proposed model, we have shown that the accuracy gained by incorporating time of patient notification and more detailed modelling of time variation is primarily realized in periods of large inflow.

A more sophisticated machine learning algorithm can possibly be applied on this forecasting problem giving more accurate forecasts. Added accuracy may, in the case of certain methods like neural nets, come with the cost of non-interpretability. In a clinical setting, as a decision support tool, interpretability of the proposed model may be valuable. For example, this makes it possible to serve information on how the expected number of arrivals is affected by time of day, the number of pre-reported patients, etc.

The approach in this study, forecasting arrivals, is only one of many potential approaches to counteracting ED crowding [1, 5]. A well-functioning ED needs to balance input, throughput and output of patients, and forecasting arrivals can be a part of an overall effort to ensure this [16]. We have demonstrated improved accuracy in forecasting over shorth time horizons. Whether this improved accuracy can make the forecasts good enough to be useful in a clinical setting should be studied further.

We recognize limitations in our study. Different health care settings and system variables will impact the usefulness of the approach. Crucially, time of patient notification must be available. This is a single center study, so the results cannot readily be generalized to other healthcare settings. We include R-code in Additional file 4, such that any ED with analogous data can test the model. Although based on manual registration, a strength of this study is that there is hardly any missing data. All ED visits are entered into the database, and all entries in the database have a time of visit registered and record creation.

## Conclusions

The objective of this study was the development of a real-time monitoring system to be used for decision support where ED arrivals are forecasted, incorporating data from prehospital services. We have proposed a model doing this based on Poison regression, with continuous handling of time variation. This method shows continuously updated forecasts with better accuracy than methods based on time variables alone, particularly for periods of large inflow.

## Additional files

Additional file 1: Derivation of approximate hazard function. (PDF 54 kb)

Additional file 2: Histogram showing the distribution of time from patient notification to arrival. (PDF 5 kb)

Additional file 3: Cyclic variation in arrivals throughout the year. (PDF 13 kb)

Additional file 4: R code for generating time series from arrival data and the prediction model. (PDF 58 kb)

## Data Availability

Data used in this study is sensitive, and will not be publicly available. R-code for the analysis is available in Additional file 4.

## Abbreviations

AIC: Akaike information criterion
ARIMA: Autoregressive integrated moving average
ED: Emergency Department
EMCC: Emergency service communication center
GLM: Generalized linear model
MAPE: Mean absolute prediction error
REC: Regional ethics committee
