# Supplementary material for: Real-time forecasting of emergency department arrivals using prehospital data
Source: BMC Emerg Med. 2019 Aug 5;19:42. doi: 10.1186/s12873-019-0256-z (PMC6683581; doi:10.1186/s12873-019-0256-z)
Supplement: Supplementary file 1 — Derivation of approximate hazard function. (PDF 54 kb) [file 12873_2019_256_MOESM1_ESM.pdf]

Derivation of approximate hazard function

We assume the time from patient record creation, t=0, to patient arrival, t=T, is distributed according to an approximate hazard function,

$$\lambda_d(t) = \frac{P(t \leq T < t + d | t \leq T)}{d}$$

We will not use the actual instantaneous hazard function, which is obtained by taking the limit d->0. Rather we use $\lambda_1(t)$, $\lambda_2(t)$ and $\lambda_3(t)$, denoting the probability of arrival within 1, 2 and 3 hours respectively, given that the patient had not having arrived at time t.

Now assume n(t) patients had records created, but were not arrived at time t, with time of record creation being $\tau_1, \tau_2, \ldots, \tau_{n(t)}$ respectively. The expected number of arrivals would be the sum of the approximate hazard functions, translated to the correct origin,

$$E[Arrivals\ within\ d\ hours] = \lambda_d(t - \tau_1) + \lambda_d(t - \tau_2) + \cdots + \lambda_d(t - \tau_{n(t)})$$

If the hazard function is approximated by a truncated polynomial of degree m,

$$\lambda_d(t) \approx a + bt + ct^2$$

then,

$$E[Arrivals\ within\ d\ hours] \approx \mathrm{a} \cdot \mathrm{n(t)} + \mathrm{b} \sum_{j=1}^{n(t)} (t - \tau_j) + \mathrm{c} \sum_{j=1}^{n(t)} (t - \tau_j)^2$$
