# Supplementary figures and images for: Real-time forecasting of emergency department arrivals using prehospital data
Source: BMC Emerg Med. 2019 Aug 5;19:42. doi: 10.1186/s12873-019-0256-z (PMC6683581; doi:10.1186/s12873-019-0256-z)

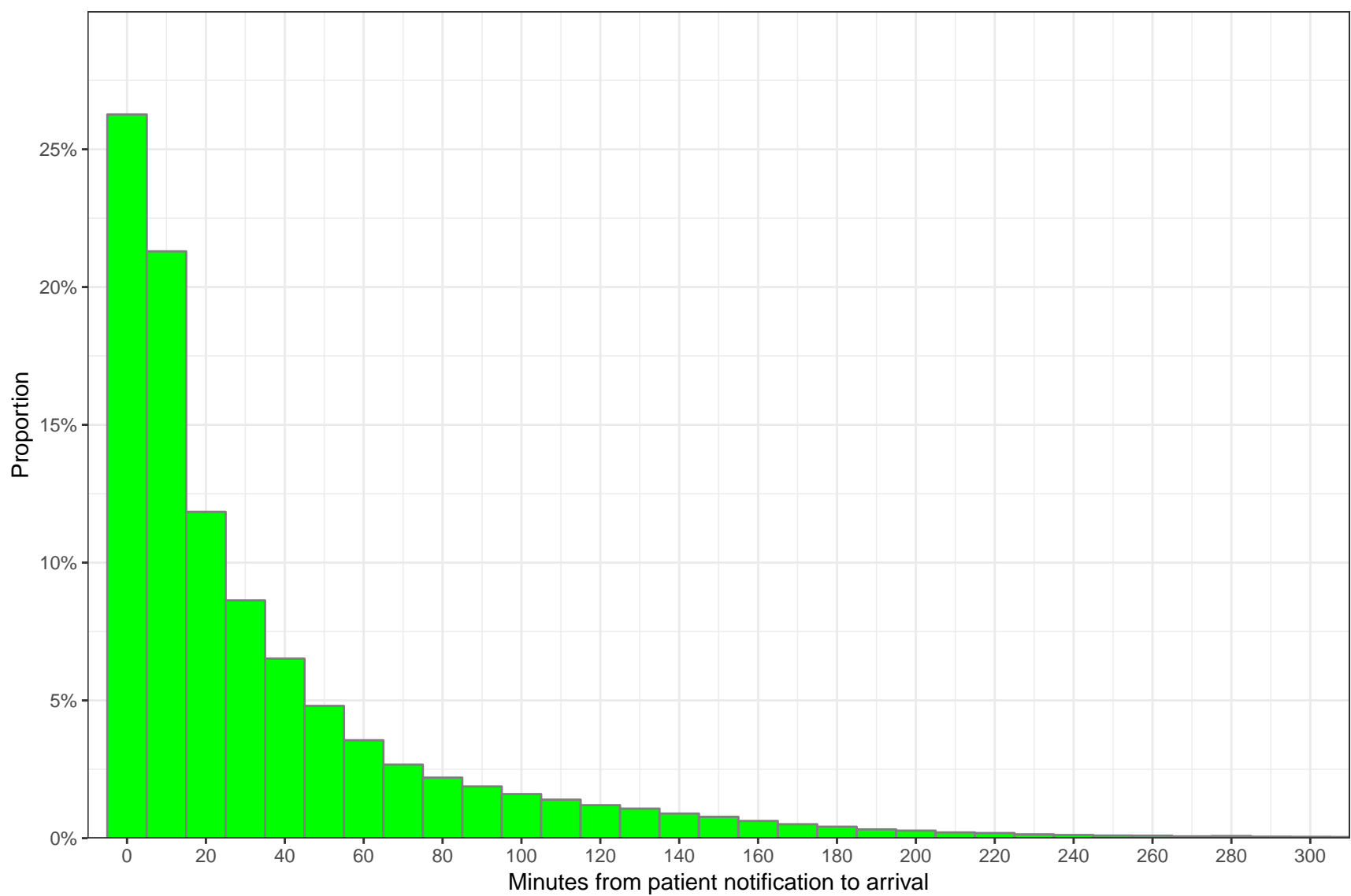

Supplement: Supplementary file 2 — Histogram showing the distribution of time from patient notification to arrival. (PDF 5 kb) [file 12873_2019_256_MOESM2_ESM.pdf]

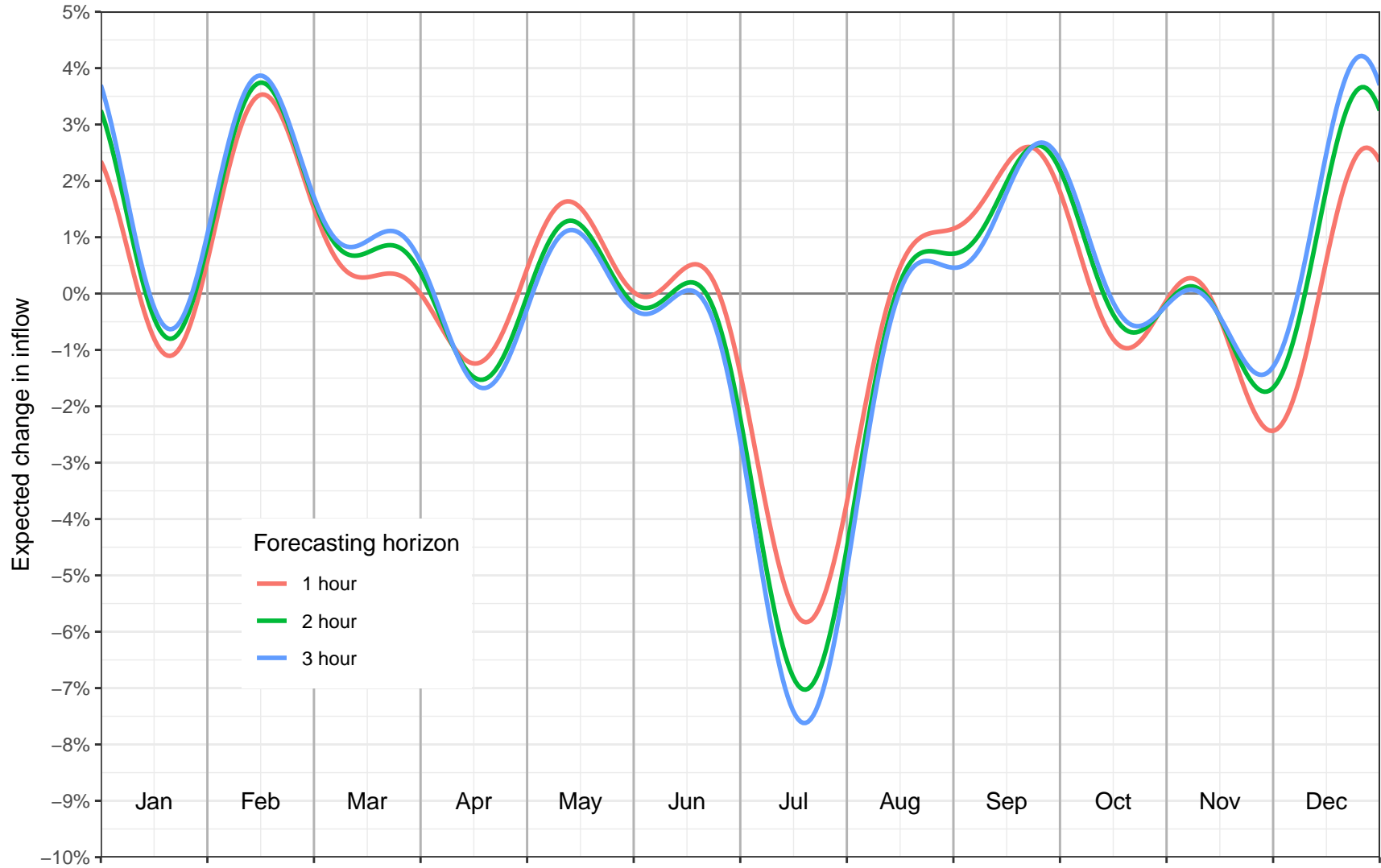

Supplement: Supplementary file 3 — Cyclic variation in arrivals throughout the year. (PDF 13 kb) [file 12873_2019_256_MOESM3_ESM.pdf]
