# Supplementary material for: Real-time forecasting of emergency department arrivals using prehospital data
Source: BMC Emerg Med. 2019 Aug 5;19:42. doi: 10.1186/s12873-019-0256-z (PMC6683581; doi:10.1186/s12873-019-0256-z)
Supplement: Supplementary file 4 — R code for generating time series from arrival data and the prediction model. (PDF 58 kb) [file 12873_2019_256_MOESM4_ESM.pdf]

R-code

```
# Input: A data frame df with variables:
#  - In, time of arrival for each patient in whole minutes since time 0
#  - Created, time of patient record creation, in whole minutes after time 0
#    If the patient was not pre-reported, Created=In

library(lubridate)

# Start and end of the period.
# All arrivals should be within these to time points
start = as.numeric(strptime("2010-01-01","%Y-%m-%d"))
stop  = as.numeric(strptime("2018-01-01","%Y-%m-%d"))

t             <- seq(start,stop, by=60)
N             <- length(t)
createdN      <- rep(0,N)
createdt      <- rep(0,N)
createdt2     <- rep(0,N)
createdt3     <- rep(0,N)
created6plus <- rep(0,N)

Arrival_1h    <- rep(0,N)
Arrival_2h    <- rep(0,N)
Arrival_3h    <- rep(0,N)

for (j in 1:nrow(df)){
  # For each patient, mark as arriving 1,2 and 3 hours in advance
  ind_1h              <- (df$inn[j]-60):(df$inn[j]-1)
  ind_2h              <- (df$inn[j]-2*60):(df$inn[j]-1)
  ind_3h              <- (df$inn[j]-3*60):(df$inn[j]-1)
  Arrival_1h[ind_1h] <- Arrival_1h[ind_1h]+1
  Arrival_2h[ind_2h] <- Arrival_2h[ind_2h]+1
  Arrival_3h[ind_3h] <- Arrival_3h[ind_3h]+1

  # For pre-reported patients
  if (df$In>df$Created){
    ind_c         <- df$Created[j]:(df$In[j]-1)
    n             <- length(ind_c)
    # If pre-reported for more than 6 hours,
    # leave out of time calculation, but
```

```
    if (n>6*60){
      created6plus[ind_c[(6*60+1):n]] = created6[ind_c[(6*60+1):n]]+1
      ind_c  <- ind_c[0:(6*60)]
      n      <- length(ind_c)
    }
    # Number of patients pre-reported at any time,
    # and sum of time (+^2 and ^3) since creation for all such patients
    createdN[ind_c]  <- nMeldt[ind_c]+1
    createdt[ind_c]  <- Meldtt[ind_c]+(1:n)
    createdt2[ind_c] <- Meldtt2[ind_c]+(1:n)^2
    createdt3[ind_c] <- Meldtt3[ind_c]+(1:n)^3
  }
}
data = data.frame(t,created,createdt,createdt2,createdt3,created6plus)

# Counting arrivals in the previous 1,2 and 3 hours
data$prev_1h             <- rep(0,N)
data$prev_2h             <- rep(0,N)
data$prev_3h             <- rep(0,N)
data$prev_1h[(60+1):N]   <- data$Arrival_1h[1:(N-60)]
data$prev_2h[(60*2+1):N] <- data$Arrival_1h[1:(N-60*2)]
data$prev_3h[(60*3+1):N] <- data$Arrival_1h[1:(N-60*3)]

# Time corrected for daylight savings time
data$t_dst <- data$t + 60*60*as.numeric(dst(as.POSIXct(data$t,origin='1970-01-01')))

# Period of yearly and weekly variation
Ty               = 60*60*24*365/2/pi
Tw               = 60*60*24*7/2/pi

# Fit the model using GLM
# Note that the variable Holiday shoulde be initialized with
# three levels: no holiday, holiday, and day after holiday
reg_1h =  glm(Arrival_1h ~
                sin(t_dst/Tw)  + cos(t_dst/Tw)+
                sin(2*t_dst/Tw) + cos(2*t_dst/Tw)+
                sin(3*t_dst/Tw) + cos(3*t_dst/Tw)+
                sin(4*t_dst/Tw) + cos(4*t_dst/Tw)+
                sin(5*t_dst/Tw) + cos(5*t_dst/Tw)+
                sin(6*t_dst/Tw) + cos(6*t_dst/Tw)+
                sin(7*t_dst/Tw) + cos(7*t_dst/Tw)+
```

```
  sin(8*t_dst/Tw) + cos(8*t_dst/Tw)+
  sin(9*t_dst/Tw) + cos(9*t_dst/Tw)+
  sin(10*t_dst/Tw) + cos(10*t_dst/Tw)+
  sin(11*t_dst/Tw) + cos(11*t_dst/Tw)+
  sin(12*t_dst/Tw) + cos(12*t_dst/Tw)+
  sin(13*t_dst/Tw) + cos(13*t_dst/Tw)+
  sin(14*t_dst/Tw) + cos(14*t_dst/Tw)+
  sin(15*t_dst/Tw) + cos(15*t_dst/Tw)+
  sin(16*t_dst/Tw) + cos(16*t_dst/Tw)+
  sin(17*t_dst/Tw) + cos(17*t_dst/Tw)+
  sin(18*t_dst/Tw) + cos(18*t_dst/Tw)+
  sin(19*t_dst/Tw) + cos(19*t_dst/Tw)+
  sin(20*t_dst/Tw) + cos(20*t_dst/Tw)+
  sin(21*t_dst/Tw) + cos(21*t_dst/Tw)+
  sin(22*t_dst/Tw) + cos(22*t_dst/Tw)+
  sin(23*t_dst/Tw) + cos(23*t_dst/Tw)+
  sin(24*t_dst/Tw) + cos(24*t_dst/Tw)+
  sin(t/Ty)  + cos(t/Ty)+
  sin(2*t/Ty) + cos(2*t/Ty)+
  sin(3*t/Ty) + cos(3*t/Ty)+
  sin(4*t/Ty) + cos(4*t/Ty)+
  sin(5*t/Ty) + cos(5*t/Ty)+
  sin(6*t/Ty) + cos(6*t/Ty)+
  sin(7*t/Ty) + cos(7*t/Ty)+
  sin(8*t/Ty) + cos(8*t/Ty)+
  t+I(t^2)+Holiday+prev_1h+prev_2h+prev_3h+
  createdN+createdt+createdt2+createdt3+created6plus,
data=data,family=poisson(link='log'))
```
